# John C. Storey, M.D., D.D.S.

**Published:** 1897-05

**Authors:** 


### Obituary.

### JOHN C. STOREY, M.D., D.D.S.

Died, Wednesday, March 17, 1897, at 11.30 P.M., John C. Storey, M.D., D.D.S., aged sixty-one.

Dr. Storey died of congestion after an illness of only a few hours. He was engaged, when seized with the fatal attack, in preparing the April number of the *Texas Dental Journal*, of which he was editor.

Dr. Storey was born in Green County, Alabama, in 1836, and his father, Dr. John C. Storey, was one of the pioneers of that State. He was graduated from the Atlanta Medical College in 1857, and practised his profession in Green County from 1857 to 1860, and then removed to Louisiana. At the beginning of the war he enlisted in the Nineteenth Louisiana Infantry, and in 1862 was discharged on account of ill health. He afterwards re-enlisted as assistant surgeon, and from this time to the close of the war he was busily engaged in caring for the sick and wounded. At the close of the war he married Miss Wiley, daughter of Rev. Dr. E. E. Wiley, of Emery, Virginia. Four children were born of this union,—John E., Clarence L., Virginia E., and Theodora J. Mrs. Storey died June 27, 1891, and her remains were interred in Trinity

Cemetery. In 1867, Dr. Storey entered the Baltimore College of Dental Surgery and was graduated in 1869. In 1875 he came to Dallas, and has resided here continuously since that time. He was a member of the Southern Dental Association and of the Texas Dental Association, and had served as president of each of these organizations.

Dr. Storey was a member of the Presbyterian Church, having joined that denomination almost thirty years ago.

He was also a member of Camp Sterling Price, United Confederate Veterans.

The funeral services were conducted by his pastor, the Rev. Dr. W. M. Anderson, in the First Presbyterian Church, at eleven o'clock Saturday morning, March 20.—*Texas Dental Journal.*

